# Understanding the Effectiveness of Genomic Prediction in Tetraploid Potato

**DOI:** 10.3389/fpls.2021.672417

**Published:** 2021-08-09

**Authors:** Stefan Wilson, Chaozhi Zheng, Chris Maliepaard, Han A. Mulder, Richard G. F. Visser, Ate van der Burgt, Fred van Eeuwijk

**Affiliations:** ^1^Biometris, Wageningen University & Research Centre, Wageningen, Netherlands; ^2^Plant Breeding, Wageningen University and Research, Wageningen, Netherlands; ^3^Wageningen University and Research Animal Breeding and Genomics Centre, Wageningen, Netherlands; ^4^Solynta, Wageningen, Netherlands

**Keywords:** tetraploid potato, genotype by sequencing, genomic prediction, genome wide association study, non-additive effects

## Abstract

Use of genomic prediction (GP) in tetraploid is becoming more common. Therefore, we think it is the right time for a comparison of GP models for tetraploid potato. GP models were compared that contrasted shrinkage with variable selection, parametric vs. non-parametric models and different ways of accounting for non-additive genetic effects. As a complement to GP, association studies were carried out in an attempt to understand the differences in prediction accuracy. We compared our GP models on a data set consisting of 147 cultivars, representing worldwide diversity, with over 39 k GBS markers and measurements on four tuber traits collected in six trials at three locations during 2 years. GP accuracies ranged from 0.32 for tuber count to 0.77 for dry matter content. For all traits, differences between GP models that utilised shrinkage penalties and those that performed variable selection were negligible. This was surprising for dry matter, as only a few additive markers explained over 50% of phenotypic variation. Accuracy for tuber count increased from 0.35 to 0.41, when dominance was included in the model. This result is supported by Genome Wide Association Study (GWAS) that found additive and dominance effects accounted for 37% of phenotypic variation, while significant additive effects alone accounted for 14%. For tuber weight, the Reproducing Kernel Hilbert Space (RKHS) model gave a larger improvement in prediction accuracy than explicitly modelling epistatic effects. This is an indication that capturing the between locus epistatic effects of tuber weight can be done more effectively using the semi-parametric RKHS model. Our results show good opportunities for GP in 4x potato.

## Introduction

Cultivated potato (*Solanum tuberosum* L.) is one of the most consumed food crops in the world, behind only rice and wheat (Birch et al., 2012). Since its discovery over 500 years ago, breeders have selected and hybridised this crop to adapt to various environmental conditions and satisfy numerous market desires. With its large genetic diversity, this was easily achieved making potato one of the most versatile food crops. Most of the environmental and market class adaptations, as well as genetic gains for simple traits, have been attained via phenotypic selection, which may take 10–12 years until a new cultivar is introduced (Jansky, 2009; Endelman et al., 2018). However, there has been limited progress for more quantitative traits with lower heritabilities, for example yield Jansky (2009). Genomic prediction (GP), where phenotypes are regressed on marker profiles (Bernardo, 1996; Whittaker et al., 2000; Meuwissen et al., 2001), allows for the early selection or discarding of favourable or unfavourable hybrids, and therefore significantly speeds up the breeding cycle (Hickey et al., 2017).

Genomic prediction has seen more application in animal breeding in comparison to plant breeding and has rarely been applied to polyploid species until recently. Cultivated potato is an autotetraploid, and the patterns of inheritance in autotetraploids are more complicated than diploids and allotetraploids (Gallais, 2003; Garcia et al., 2013; Dufresne et al., 2014), hence the reason for the smaller number of GP studies among these species. Despite the obstacles, GP has recently been put to use in a number of autopolyploid crops including alfalfa (Annicchiarico et al., 2015), potato (Habyarimana et al., 2017; Sverrisdóttir et al., 2017; Enciso-Rodriguez et al., 2018; Endelman et al., 2018; Amadeu et al., 2020), blueberry de Bem Oliveira et al. (2019), Amadeu et al. (2020), and tetraploid ryegrass Guo et al. (2018).

Despite the common theme of past studies, in that they look at GP in autopolyploids, they differ in more ways than just the species they focus on. This study intends to merge some of the principles used in previous studies. Genotype by sequencing (GBS) has been utilised previously in the study of GP of autopolyploid crops (Annicchiarico et al., 2015; Sverrisdóttir et al., 2017; Guo et al., 2018), and will be implemented in this study as the method for investigating DNA variation. One difficulty encountered in quantitative genetics for polyploids is the determination of allele dosage. Recent studies have investigated methods to deal with this problem (Endelman et al., 2018; Guo et al., 2018; de Bem Oliveira et al., 2019) by looking directly at allele frequencies and refraining from performing discrete genotype calling. This study also directly examines allele frequencies, but uses a probabilistic approach for determining the most likely dosage based on allele frequency ratios.

Statistical models used for GP face the scenario where *n* < < *p*, therefore penalties are introduced for reliable estimation of marker effects, which require assumptions on the parametric distribution of these marker effects (Piepho, 2009). The most common GP model is known as GBLUP (Genomic best linear unbiased predictor), a mixed model, where the relationship between cultivars is used as input, and is equivalent to using a ridge regression penalty with an assumed normal distribution for marker effects (Piepho, 2009). A relationship matrix can be derived assuming additive effects and non-additive effects (dominance and epistasis). We investigate the impact of explicitly accounting for non-additive effects (Enciso-Rodriguez et al., 2018; Endelman et al., 2018; Amadeu et al., 2020) vs. implicitly modelling these non-additive effects using the semi-parametric Reproducing Kernel-Hilbert Space (RKHS) model (Gianola and van Kaam, 2008; Habyarimana et al., 2017). Another relationship matrix has been proposed for autotetraploids, that assumes separate genotype effects for each marker (Slater et al., 2016) which also implicitly captures non-additive effects and is included in this study. Bayesian models are also included in this study, to compare the impact of different prior assumptions on the distribution of marker effects (Pérez and de los Campos, 2014).

For GP, there is no “one-size-fits-all” model that works best, and instead the performance of models depends primarily on trait architecture (de los Campos et al., 2013). Unlike many GP studies, we extend this study to include a Genome Wide Association Study (GWAS), to describe the architecture of each trait and explain the differences in the performance of the various GP models. Applying GWAS to markers coded for different types of dominance (Rosyara et al., 2016), we attempt to identify the source of dominance effects, for those traits that were more accurately predicted with GP models that included non-additive effects. GWAS will also reveal the level of association between our markers and a particular trait, to understand why a GP model that estimates marker effects performs better than a model that estimates genotype effects or vice versa.

We aim to demonstrate the feasibility of GP in autotetraploid potato in this proof-of-concept study. Using four traits and GBS marker data, various modelling strategies will be compared to uncover the model or models most suitable for a given trait. To comprehend the relationship between a trait and its most suitable model, a GWAS is used to describe the genetic architecture of the traits, providing some insight as to why some modelling strategies might work better for particular traits.

## Materials and Methods

### Plant Materials

A diversity panel of 147 tetraploid potato cultivars, including recent Dutch breeding material were chosen for this study. This subset of cultivars are representative of the worldwide commercial potato germplasm and were selected based on criteria such as: phenotypic diversity of important traits, country of origin, market category (chip and French fry processing, cooking and starch varieties), year of commercial introduction, and availability of the cultivars. Some of these varieties were analysed in previous studies that used similar criteria for selection (D'hoop et al., 2008, 2014). Propagation was done by two Dutch breeding companies, one of which had also performed phenotyping and collecting the biological material needed for genotyping.

#### Genotypic Information

DNA material (100 ng) was digested with ten units of EcoT22 (Clontech) and incubated at 37°C for 2 h and then heat killed. Samples were then ligated with 640 units of T4 ligase (NEB) and phased adaptors with TGCA overhangs at 22°C for 1 h and heat killed. The ligated samples were diluted in the ratio 1:10 with water, and then amplified for 18 cycles to add barcodes. Barcoded libraries were SPRI purified, quantified, and pooled in groups of 48 samples. Pooled samples were SPRI purified, quantified, and diluted to 2 nM for sequencing on the Illumina HiSeq 2500 using single-end 1 × 100 reads. Sequence reads were mapped against the potato reference genome sequence of DM v4.04, including the chloroplast and mitochondrial sequences using Burrows-Wheeler Aligner 0.7.12. After the removal of monomorphic markers, those with more than two alleles and markers from repetitive regions of the genome, 870 thousand bi-allelic markers were available for further filtering. Markers with minor allele frequency <0.01 and those with read depths <10 or >100 were removed. From the remaining markers, the posterior probability of allele dosage, conditional on both allele counts and sequencing error, was calculated (see Supplementary Material for more details). This will be referred to as the genotype assignment probability. Tetraploid genotypes can belong to either of the classes AAAA, AAAB, AABB, ABBB, BBBB, where “A” and “B” are the reference and alternative allele, respectively. If there is an equal amount of counts for both alleles we would infer the genotype to be AABB (see example in Table 1). Similar methodology is applied in the PolyOrigin software (Zheng et al., 2020).

**Table 1:** Two examples of genotype probabilities based on allele counts.

**Allele count**		**Genotype probabilities**				
**Reference**	**Alternative**	**AAAA**	**AAAB**	**AABB**	**ABBB**	**BBBB**
15	13	0	0.05	0.94	0.01	0
15	0	0.99	0.01	0	0	0

**Figure 1:**
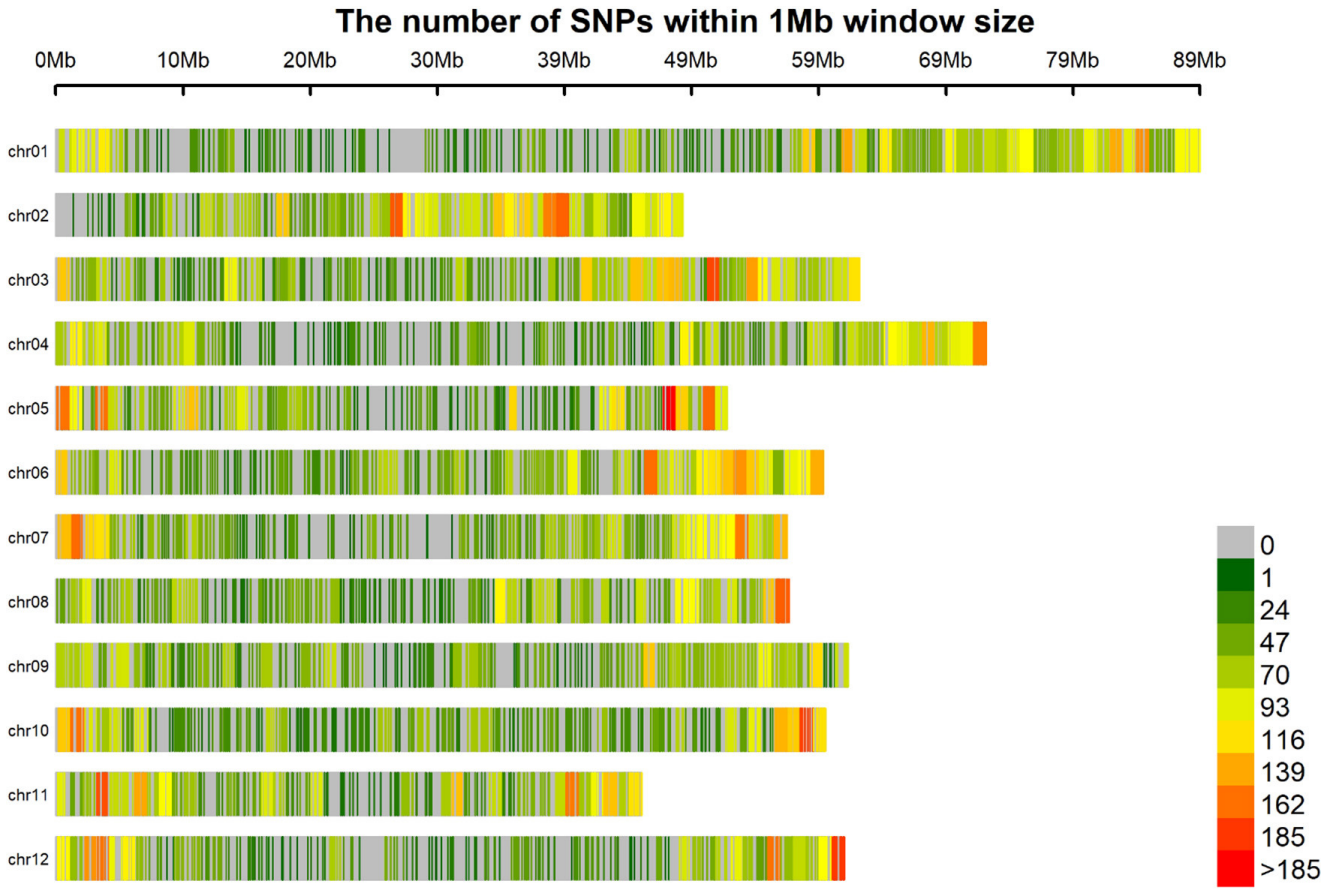
Marker density of 39 K markers.

Genotype assignment probabilities were used as a filter criterion. For each individual, markers were removed when the highest genotype assignment probability was below a threshold. Stricter thresholds created more missing information and decreased the number of markers, since markers without information for more than 25% of the individuals were removed. Allele dosage was then determined as the dosage with maximum genotype probability. Probability thresholds of 0.85, 0.75, and 0.5 resulted in marker matrices of 19, 26, and 39 thousand markers, respectively. Using an additive GBLUP model, a preliminary GP analysis was performed to decide which marker matrix should be used as there may be a trade-off between the quantity and quality of markers. In almost all cases, the 39 K marker matrix gave the most accurate predictions and will henceforth be used for all analyses (Supplementary Figure 1). The larger number of markers lends itself to a more complete coverage of the genome (Figure 1).

Although linkage disequilibrium (LD) was not calculated in this study, it was calculated for an overlapping panel of tetraploid potato (Vos et al., 2017). In that study, it was found that LD falls quickly and suggested 40 K markers were needed for good coverage of the tetraploid potato genome for GWAS, which is comparable to the number of markers used here.

For all analyses performed in this study, we begin with genotype information contained in the marker matrix (X), with 147 rows and 39,000 columns. Each element of X gives the discrete count of alternative alleles (0, 1, 2, 3, 4) assigned by genotype probabilities, at a given marker position for a given cultivar. When these counts are entered in a design or relationship matrix and a single parameter is estimated to quantify the dependence of the phenotype on the allele count, then this implies that marker effects are additive.

For imputing the missing marker information, the mode was used. This was compared to mean imputation using the 39 K marker set mentioned above and a GBLUP model. The GP accuracies resulting from marker matrices imputed with the mode were slightly higher than those imputed from the mean.

#### Phenotypic Information

Field trials were performed in 2017 and 2018 at three locations: Spain, Poland, and the Netherlands. Seed tubers were planted in plots consisting of eight plants. A row-column resolvable design was implemented with two complete blocks, and varieties dispersed across the field using latinisation over rows and columns (Piepho et al., 2015). Checks of one particular variety were uniformly distributed throughout the trial in order to detect and correct for spatial trends. Randomisation was performed using the package DiGGer (Coombes, 2009) executed with the software R (R Core Team, 2019), where all analyses were conducted with this study.

Four traits will be discussed in this study: plot tuber weight (kilograms), plot tuber count (number of tubers), mean tuber length (millimetres), and dry matter content (percentage). Adjusted means were calculated by correcting for row and column trends, as well as block effects using the model:

(1) $$\begin{array}{r} y=block+rowinblock+colinblock+G+\epsilon , \end{array}$$

where *y* is a vector of phenotypic observations. Equation (1) allows us to adjust for field trends (from blocks, rows within blocks, and columns within blocks) and extract the best linear unbiased estimate (BLUE) of each genotype (G). Complete blocks were used in each trial therefore a fixed term for the block effect is suitable in the statistical model. Rows and columns within blocks were incomplete and therefore treated as random effects having normal distributions as follows: $row\;\sim \;N\left(0,\sigma_{row}^{2}\right)$ and $col\;\sim \;N\left(0,\sigma_{col}^{2}\right)$ where $\sigma_{row}^{2}$ and $\sigma_{col}^{2}$, are row and column variances, respectively. Other non-genetic factors are captured in the random term ϵ that is assumed to be normally distributed as $\epsilon \;\sim \;N\left(0,\sigma_{\epsilon }^{2}\right)$ where the residual variance is represented by $\sigma_{\epsilon }^{2}$.

For investigating genotype by environment interaction (GxE), the BLUEs from the six trials (three locations, 2 years) will be useful, however for this application of GP, we require one vector of observations for a given trait, as if they came from one single environment. To consolidate our six phenotypic values, we again calculated the adjusted means of each genotype, after correcting for the effect of different trials using Equation (2).

(2) $$\begin{array}{r} y=trial+G+\epsilon , \end{array}$$

where *y* are the BLUEs calculated from Equation (1), and ϵ captures the variation from the interaction between genotype and trial as well as within trial error variation. Equation (2) is an across trial model, while Equation (1) was used for within trial analyses. This could have been combined in one statistical model, but for future GxE applications, and the ability to carefully assess each trial for outliers, it was conducted in two steps. A comparison of BLUEs calculated from the method described here vs. one single model was done and the results were the same. The BLUEs from Equation (2) will be used as the response variable for GP analyses going forward. This study is therefore a two-step analysis since phenotypic adjusted means and GP are done with separate models. ASREML (Butler, 2009) was used to conduct all phenotypic analyses.

#### Heritability

To have an understanding of how much phenotypic variation can be attributed to between-genotype variation, broad-sense heritability was calculated. Using the BLUEs from Equation (1) as the response variable, we apply the following model across our three locations (*L*) and 2 years (*T*):

y=L+T+LT+G+GL+GT+ϵ

Using the random terms of this model, highlighted in bold font, we can isolate the variability that is caused genetically from the variability that is caused from genotype by location and genotype by year interactions (*GL, GT*). The BLUEs from Equation (1) give only one value for each genotype per trial (year and location combined), for this reason our error term (ϵ) in the heritability equation captures the variation from the three way interaction of genotype, location, and year. Averaging a genotypic effect across multiple trials without including marker information, is closer to an estimation of repeatability than heritability (Falconer et al., 1996), but for now we shall use traditional nomenclature. Heritability was calculated from the variance components as:

$$H^{2}=\frac{\sigma_{g}^{2}}{\sigma_{g}^{2}+\frac{\sigma_{gL}^{2}}{l}+\frac{\sigma_{gT}^{2}}{t}+\frac{\sigma_{\epsilon }^{2}}{l\times t}},$$

where *l* and *t* represent the total numbers of locations and years. Variation due to genotype by location and genotype by year interactions are represented by the terms $\sigma_{gL}^{2},\sigma_{gT}^{2}$, respectively, while $\sigma_{g}^{2}$ represents genetic variance. The term $\sigma_{\epsilon }^{2}$ is the variance of the three way interaction of genotype by year by location, and contains genetic signal alongside within trial variation.

### Prediction Models

For GP, many types of statistical models are applicable; those that perform shrinkage vs. those that perform variable selection which is dependent on the assumed distribution of marker effects, and those models that account for non-additive effects in various ways.

- **Additive GBLUP:**
  (3) $$\begin{array}{r} y=\mu +Z_{a}a+\epsilon , \end{array}$$
  where *y* is a vector of phenotypic values, μ is the overall mean, *Z*_*a*_ is a design matrix that relates the observations to genomic values and *a* is a vector of random additive genetic values with distribution $a\;\sim \;N\left(0,G_{A}\sigma_{a}^{2}\right)$. The additive genetic variance is given by $\sigma_{a}^{2}$, while ϵ is the vector of residual and non-modelled genetic effects, assumed to be normally distributed $\epsilon \;\sim \;N\left(0,\sigma_{\epsilon }^{2}\right)$, with variance denoted by $\sigma_{\epsilon }^{2}$. *G*_*A*_ is the additive genomic relationship matrix (from allele dosages) based on the work of VanRaden (2008) and extended by Ashraf et al. (2016). The calculation of this additive genomic relationship matrix is applicable to autotetraploids and was constructed with the R package AGHmatrix (Amadeu et al., 2016).
- **Additive + Dominance GBLUP:**
  (4) $$\begin{array}{r} y=\mu +Z_{a}a+Z_{d}d+\epsilon , \end{array}$$
  where *y*, μ, *a*, and ϵ are the same as seen in Equation (3). *Z*_*a*_ and *Z*_*d*_ are design matrices to relate observations to additive genetic effects and dominance effects. The vector of dominance effects is indicated by *d* and follows a normal distribution: $d\;\sim \;N\left(0,G_{D}\sigma_{d}^{2}\right)$, where $\sigma_{d}^{2}$ is the dominant genetic variance. The digenic dominant relationship matrix *G*_*D*_ was built using the AGHmatrix R-package, as derived by Endelman et al. (2018).
- **Epistatic GBLUP:**
  (5) $$\begin{array}{r} y=\mu +Z_{a}a+Z_{d}d+Z_{e}e+\epsilon \end{array}$$
  Equation (5) is an extension of Equation (4), with the inclusion of a term to capture epistatic effects. *Z*_*e*_ relates the observations to the epistatic effects *e*, which follow the normal distribution, $e\;\sim \;N\left(0,G_{E}\sigma_{e}^{2}\right)$ with epistatic genetic variance $\sigma_{e}^{2}$.
  This paper considers first order epistasis (additive × additive), and to calculate *G*_*E*_, the Hadamard product of *G*_*A*_ (*G*_*A*_*#G*_*A*_) was used (Su et al., 2012; Endelman et al., 2018).
- **Full Auto-tetraploid GBLUP:**
  (6) $$\begin{array}{r} y=\mu +Zf+\epsilon , \end{array}$$
  Proposed in the paper by Slater et al. (2016) is the full auto-tetraploid model which accounts for additive and non-additive effects by assuming each genotype has its own effect. Tetraploids have five possible genotypes (AAAA, AAAB, AABB, ABBB, BBBB), therefore *f*, the vector of effects in Equation (6), has length 5*R* where *R* is the number of markers (see Table 2). These effects *f*, follow the normal distribution, $f\;\sim \;N\left(0,G_{F}\sigma_{f}^{2}\right)$ with genetic variance $\sigma_{f}^{2}$. The details for calculation of the relationship matrix *G*_*F*_ can be found in the associated literature (Slater et al., 2016), and was constructed using the AGHmatrix R-package.
- **RKHS:** The model for Reproducing Kernel-Hilbert Space (RKHS) is the same as described in Equation (3), but the random genetic values have a different distribution: $a\;\sim \;N\left(0,K\sigma_{g}^{2}\right)$. The genomic relationship matrix *G*_*A*_, is replaced by the kernel matrix, $K=exp^{-\frac{D}{\theta }}$, where D is a Euclidean distance matrix between genotypes, and θ a tuning parameter. The tuning parameter controls how fast the relationship between two genotypes decays as the distance between the corresponding pairs of marker vectors increases (Jiang and Reif, 2015) and is estimated from the data by maximizing the log-likelihood (Endelman, 2011). The genetic variance is no longer the result of allele substitution, as seen in the additive model (Equation 3) with additive genetic variance, $\sigma_{a}^{2}$. The genetic variance captured by RKHS ($\sigma_{g}^{2}$), includes additive and first order epistatic (additive × additive) effects (Gianola and van Kaam, 2008).
- **Bayesian LASSO:**
  (7) $$\begin{array}{r} y=\mu +Xb+\epsilon \end{array}$$
  The first five genomic predictions described above estimate genotypic effects, the Bayesian models however estimate marker effects. Equation (7) includes terms for phenotype (*y*), overall mean (μ), and non genetic (or unmodelled) influences plus error (ϵ). Where it differs from our previous GP models is in the term *Xb*, which directly links the marker design matrix *X*, to the marker effects *b*. The marker effects are assumed to come from a distribution and in the case of Bayesian LASSO, a double exponential (Laplace) distribution, *b* ~ *Lap*(0, λ), or alternatively:
  $$b\sim \Pi_{j=1}^{R}\frac{\lambda }{2}e^{-\lambda |b_{j}|}$$
  The λ parameter is inversely proportional to the variance of the distribution and is estimated from the data. The probability density function is multiplied across all markers (each indicated by subscript *j*), up to a total of *R* markers.
- **Bayes A:** The statistical model is similar to that seen in Equation (7), however Bayes A assumes that marker effects come from a scaled-t distribution with *v* degrees of freedom, $b_{j}\sim t_{v}\left(0,\sigma_{b}^{2}\right)$, where $\sigma_{b}^{2}$ is the variance of marker effects.
- **BAYES Cπ:** The Bayes Cπ model assumes that marker effects come from a mixture distribution where a proportion of markers (π) have zero effect and the remainder (1 − π) have non-zero effects from a normal distribution, such that:
  $$b_{j}=\{\begin{array}{ll} 0 & :\text{with probability }\pi \\ \;\sim \;N(0,\sigma_{b}^{2}) & :\text{with probability }1-\pi \end{array}$$
  Because markers are separated into either having an effect or having no effect, this model is performing marker selection. The proportion of zero effect markers π, is estimated from the data.

**Table 2:** Within marker locus coding for Full Tetraploid model (Slater).

**Genotype**	**Locus coding**				
AAAA	1	0	0	0	0
AAAB	0	1	0	0	0
AABB	0	0	1	0	0
ABBB	0	0	0	1	0
BBBB	0	0	0	0	1

*For a genotype “AAAA,” there are five columns with the first column assigned a 1 and the rest 0's*.

The three Bayesian models are better suited for traits controlled by few large effect loci, whereas the models mentioned before are better for predicting traits with many small effect loci (de los Campos et al., 2013). For all GP models, except RKHS, parameters were estimated using Bayesian statistics (Gibbs Sampler) with the package BGLR (Pérez and de los Campos, 2014), with 10,000 iterations and 2,500 iterations used as burn-in. Maximum likelihood was used to implement the RKHS model, and choose the most likely value for the tuning parameter.

### Assessing Prediction Accuracy

With 147 varieties containing both phenotypic and genotypic information, cross-validation was performed by sampling a training set of 105 individuals to train the model, and using the trained model to predict the remainder of individuals (validation set) (Wilson et al., n.d.). These 105 individuals were sampled in order to minimise the genetic distance between the training and validation sets, using a sampling method based on the coefficient of determination (Rincent et al., 2012). This training set construction procedure uses marker information in the form of a genomic relationship matrix, as well as phenotypic information to construct the training set. Prediction accuracy is defined as the Pearson correlation between the BLUEs and the predicted genotypic values, and was averaged over the 100 repetitions.

### Genome Wide Association Study (GWAS)

To suggest an explanation for the differences between GP models, a GWAS was performed to investigate the genetic architecture of the traits analysed. The proposed GP models assume different biological processes for controlling trait expression: many small effect loci vs. a few large QTLs as well as additive vs. dominant effects. For a given trait, the genetic architecture uncovered by GWAS will help explain why a particular GP model has higher prediction accuracy than another.

(8) $$\begin{array}{r} y=\mu +X\beta +g+\epsilon \end{array}$$

In Equation (8), *y* is the vector of BLUEs, μ is the overall mean. The polygenic effect is captured by the term *g*, and is distributed $g\;\sim \;N\left(0,G_{A}\sigma_{g}^{2}\right)$, where *G*_*A*_ is the same genomic relationship matrix across all chromosomes, used for the GBLUP prediction in Equation (3). The error term ϵ captures non-genetic residuals plus error, and is assumed to follow a normal distribution as seen in prior models. The term β represents the marker effect and *X* is the marker matrix containing genetic information that may be coded differently depending on the assumed type of effect (see Table 3).

**Table 3:** Marker configurations under different effect assumptions.

	**AAAA**	**AAAB**	**AABB**	**ABBB**	**BBBB**
Additive	0	1	2	3	4
Simplex dominant (B>A)	0	4	4	4	4
Duplex dominant (B>A)	0	0	4	4	4

From Table 3, we see the coding of the design matrix, where the additive effect assumes the size of the effect is proportional to the number of alternative alleles present. Simplex dominant (for the alternative allele) indicates that there are two levels for effects: when there is no alternative allele present and another for when there is at least one alternative allele. This simplex dominant configuration of allele effects corresponds with our GBLUP dominance prediction model (Equation 4). Duplex dominance means that the second level of effect occurs when at least two alternative alleles are present. Duplex dominance was not included in any GP models, however exploring the level of dominance can reveal genetic architecture information and therefore, help explain the differences between GP accuracies, allowing for expansion in future studies. For both simplex and duplex dominance, the reference allele was also regarded as the dominant allele, and therefore five different SNP design matrices (additive, simplex dominance for the reference allele, simplex dominance for the alternative allele, duplex dominance for the reference allele and duplex dominance for the alternative allele) were used in the GWAS of each trait. This analysis was done using the GWASpoly package (Rosyara et al., 2016).

The impact of population structure on the GWAS analysis was evaluated by looking at the quantile-quantile plots of the *p*-values for marker effects transformed to a log scale (−*log*_10_*p*). Not correcting for population structure will result in spurious associations, and this was investigated by a visual assessment for inflation of *p*-values.

The threshold for identifying significantly associated markers was corrected for multiple testing using the method proposed by Li and Ji (2005). This is calculated as the significance level divided by the number of effective regions ($\frac{\alpha }{N_{eff}}$), where *N*_*eff*_ is estimated from the eigen values of the marker matrix. This resulted in 222 effective regions from the 39,000 markers. For each marker effect assumption (additive, simplex dominance etc.), significant markers were extracted and used as explanatory variables, along with the first three principal components (extracted from the relationship matrix constructed on allelic dosage), in a linear regression model. The *R*^2^ statistic of this model is the fraction of the total sum of squares due to genotypic differences, that can be explained by markers (Wallace et al., 2016; Inostroza et al., 2018). For a given trait, we will be able to distinguish which effect, additive, dominance, or the effect of population structure, explains more of the phenotypic variance.

## Results

### Population Structure

**Figure 2:**
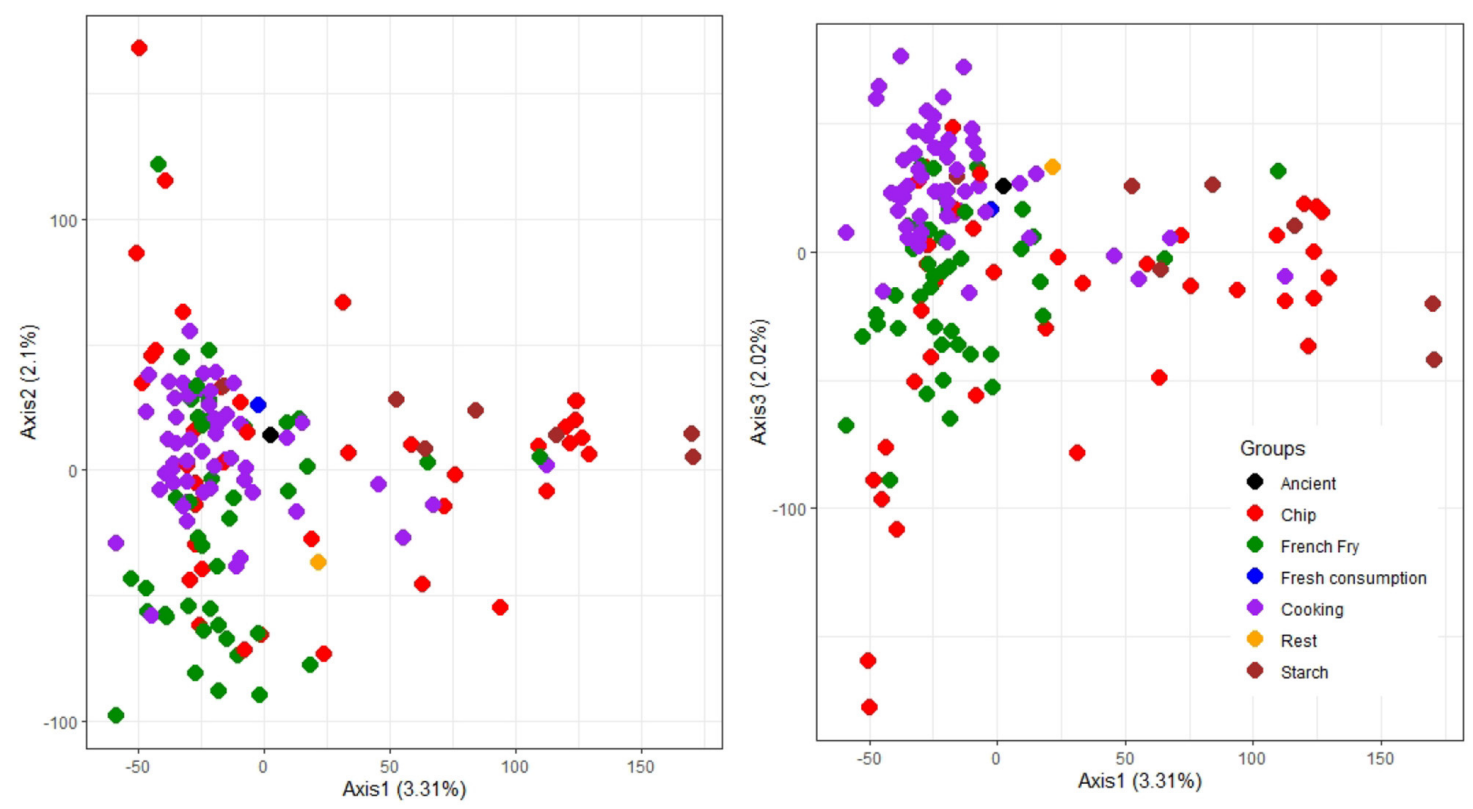
Illustration of the population structure explained by the first three Principal Components (PCA) of the entire genome, with market class membership indicated by colour.

Using the marker matrix X (described previously in the Materials and Methods) an assessment of population structure was conducted via Principal Components analysis (Figure 2), analysis of molecular variance (AMOVA) and Wright's *F*_*ST*_ statistic (Table 4). A list of the seven distinct market classes are as follows, with the number of individuals belonging to each class given in parentheses: ancient (1), chip processing (39), French fry processing (42), fresh consumption (1), cooking (56), starch (7), and the rest (1).

**Table 4:** *F*_*ST*_ statistic between sub-populations.

***F*_*ST*_**	**Cooking**	**French fry**	**Chip**
French fry	0.0088		
Chip	0.0116	0.0098	
Starch	0.0323	0.0341	0.0130

*Numbers close to zero indicate populations that are more genetically similar*.

Figure 2 illustrates that there is a lack of separation between market classes. For *F*_*ST*_ and AMOVA calculations, the three small market classes were not included as they did not meet the requirement of minimum population size for these analyses (Willing et al., 2012; Nazareno et al., 2017). Population classifications contributed only 6.7% of the total molecular variation according to the results of AMOVA, further supporting what we see in Figure 2. The four major market classes showed very little separation with *F*_*ST*_ values close to zero (Table 4), indicating that these sub-populations are genetically similar. The starch market class is closer to the chip processing group than the cooking and French fry processing classes as shown in Table 4, and illustrated in Figure 2.

All population structure analyses were performed using the R packages StaMPP (Pembleton et al., 2013) and Adegenet (Jombart and Ahmed, 2011), because of their suitability for polyploid population genetics (Dufresne et al., 2014).

### Phenotypic Analysis

**Figure 3:**
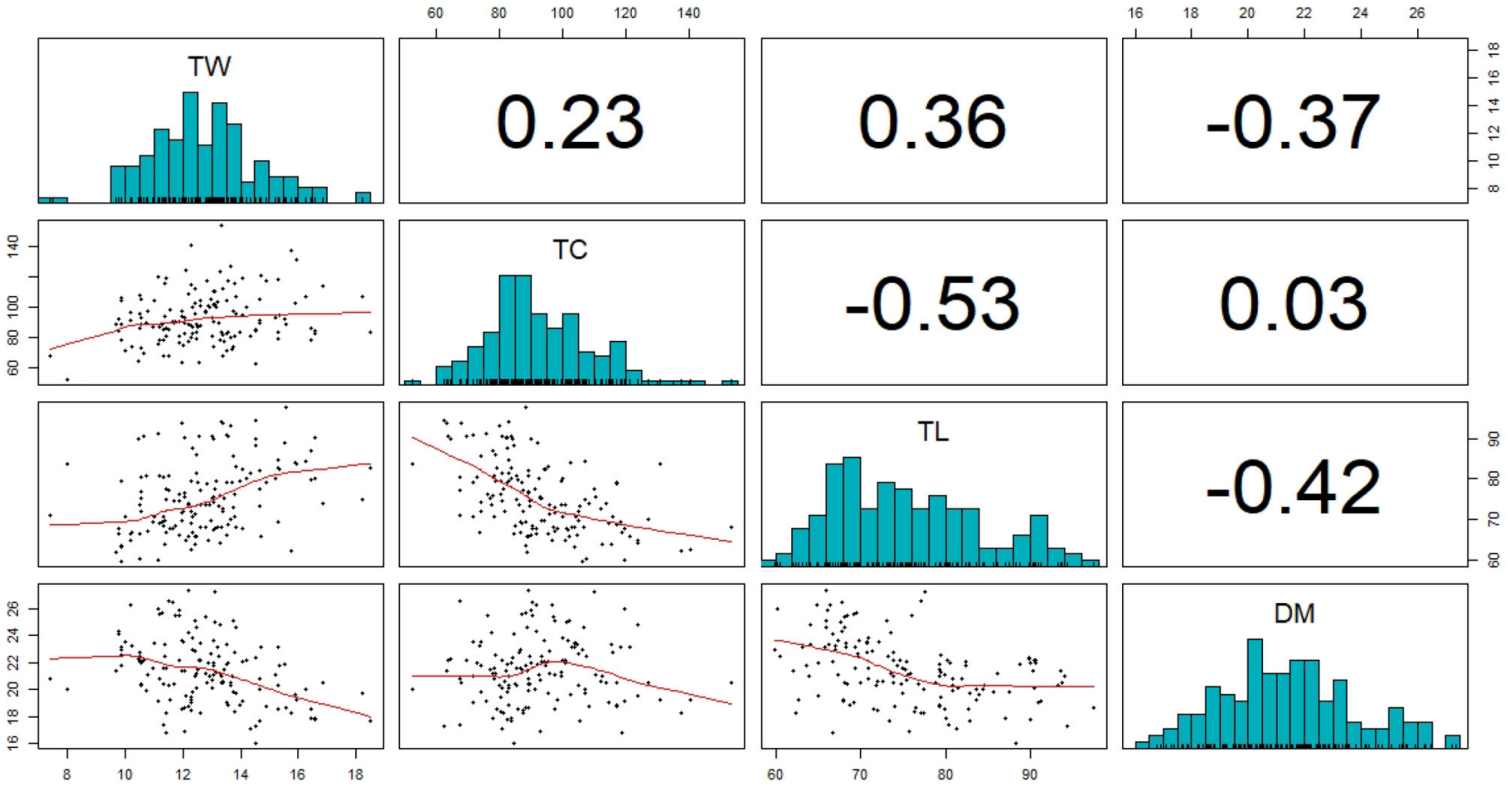
Distribution and correlation between the four analysed traits: Tuber Weight (TW), Tuber Count (TC), Tuber Length (TL), Dry Matter (DM).

Phenotypes were first adjusted for local trends within each trial as seen in Equation (1). At this level of analysis, outliers were detected and removed and the extracted BLUEs were then pooled across all trials as described in the Materials and Methods section. The resulting distributions and correlations between phenotypic values can be seen in Figure 3.

Broad-sense heritability was calculated for tuber weight, tuber count, tuber length, and dry matter resulting in *H*^2^ values of 0.78, 0.79, 0.91, and 0.96, respectively. These heritability estimates are quite high and most likely because of the repeated trials at three locations and 2 years.

### Genomic Prediction

**Figure 4:**
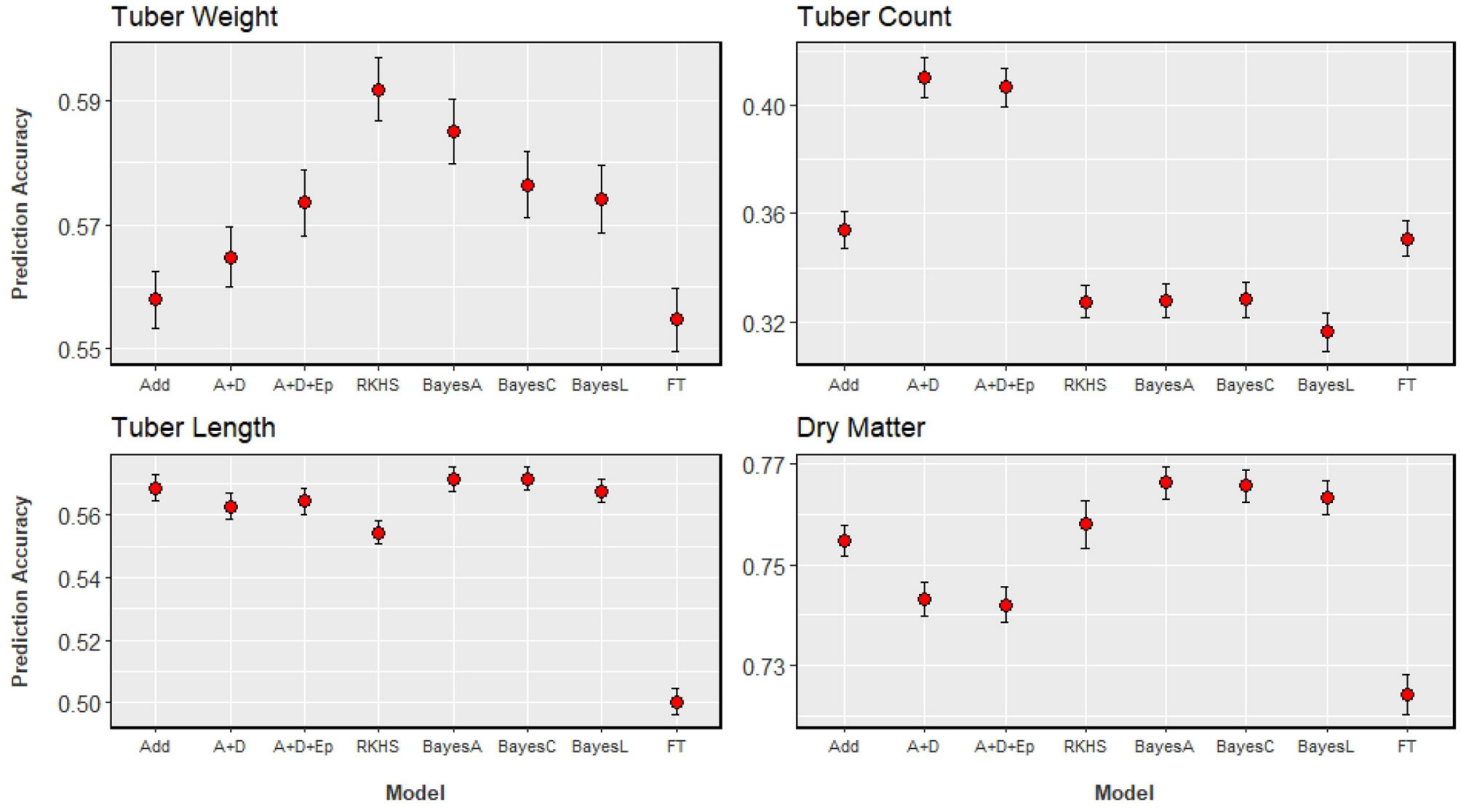
GP results of the four analysed traits, with prediction accuracy on the y-axis, and the x-axis indicating the model used: Add (GBLUP with additive genomic relationship matrix), A+D (GBLUP with additive and dominance relationship matrices), A+D+Ep (GBLUP with additive, dominance, and epistatic relationship matrices), RKHS (Reproducing Kernel-Hilbert Space model), BayesC (Bayes Cπ model), BayesL (Bayesian LASSO model), FT (Full Tetraploid as proposed by Slater et al., 2016). Standard errors of estimates are illustrated with the bars around the points.

The results of GP analyses on the four traits, compared across eight statistical models can be seen in Figure 4. Accuracies ranged from 0.32, when tuber count was predicted with a Bayesian LASSO model, to 0.77 when dry matter content was predicted with a Bayes-A model. With the highest heritability, it is not surprising that dry matter has the highest prediction accuracy. Tuber length was predicted more accurately than tuber count, and this corresponds with the ordering of their heritability estimates. The trait with the second highest prediction accuracy was tuber weight, which was unexpected as it had the lowest heritability, and was the only trait that did not agree with the order of heritability estimates.

There is no clear ranking of model performance across all traits, however Figure 4 allows us to observe some trends. The three Bayesian models, that differ in their assumed distribution of marker effects, show little difference between them across all traits (differences of at least 0.03 will be considered relevant). Bayesian models were among the better performing models, for those traits that were also predicted well by an additive GBLUP model. Extending an additive model to include dominance or dominance + epistasis did not significantly improve prediction accuracies for the traits analysed, except for tuber count. For dry matter content and tuber length, the addition of these non-additive effects decreased prediction accuracy, but these decreases were not relevant. With tuber count again being the exception, the performance of the RKHS model was comparable to the model that best predicted a given trait. The full tetraploid model (FT) was generally outperformed by all the other models, more so for dry matter content and tuber length.

Model ranking can better be assessed on a per trait basis, as the best performing model depends on the trait analysed. Figure 4 shows that models that directly estimate marker effects are the most suitable for predicting dry matter. Tuber length can be predicted efficiently with either an additive GBLUP or one of the Bayesian models. Tuber weight prediction appears to benefit from modelling non-additive effects, but the source of that effect is not completely clear. There is a small increase in prediction accuracy as we move from additive, to an additive-dominant model, to a model that includes additive, dominance and additive × additive epistatic effects. This trend could suggest the presence of a non-additive effect not explicitly modelled by the GBLUP models, such as an effect that is of a higher order than the additive × additive epistatic interaction. The RKHS model produced the highest accuracies for this trait, and is unique among the models tested as all other models are parametric while the RKHS model is semi-parametric. The RKHS model captures the same first order epistasis as the parametric model, however it gives the most noticeable improvement in comparison to the standard additive GBLUP model (from 0.56 to 0.59). For predicting tuber count, extending the additive GBLUP model to include dominance effects improved the accuracy of prediction by 17%, from 0.35 to 0.41, which we consider as a relevant change. The explicit modelling of this particular non-additive effect is clearly beneficial for the prediction of this trait, more so than any other trait analysed.

An additional result from the Bayes Cπ model is the fraction of markers selected because of their potential QTL effects. For dry matter 0.27 markers were selected while the for tuber weight, half (0.5) of the markers were selected. The proportion of selected markers for tuber length and count was 0.35 and 0.34, respectively. This gives an idea of trait architecture which is investigated further in the next section.

### GWAS

**Figure 5:**
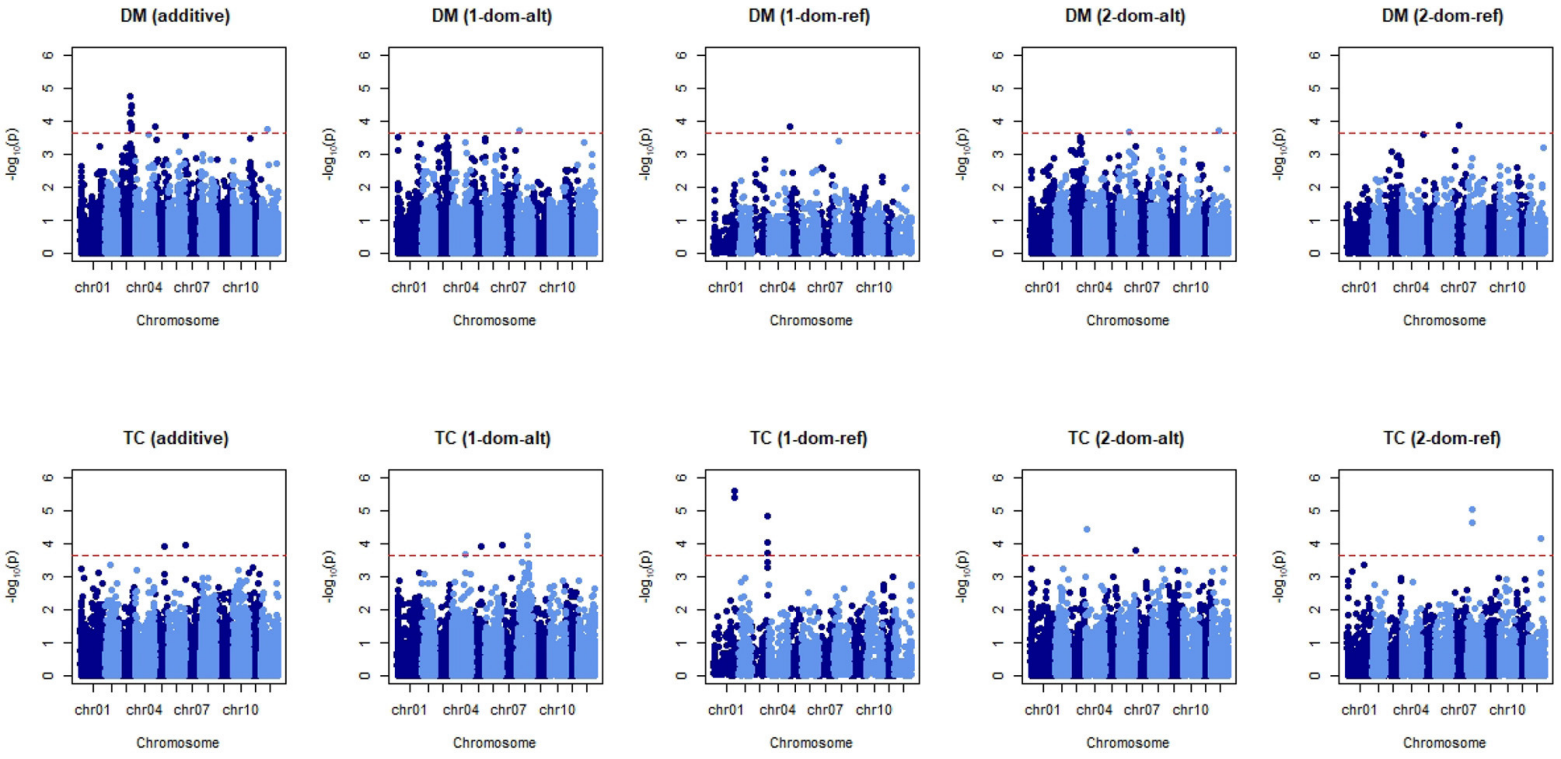
Manhattan plots for dry matter (DM) and tuber count (TC). Five marker matrices were tested: additive, simplex dominant in favour of the alternative allele and reference allele (1-dom-alt and 1-dom-ref, respectively), duplex dominant in favour of the alternative allele and reference allele (2-dom-alt and 2-dom-ref, respectively). The red horizontal line indicates the threshold for significant markers.

Trait architecture is responsible for the particularity between the accuracy of a GP statistical model and the trait analysed. To uncover some of the underlying genetic behaviour responsible for the expression of our four traits, Equation (8) was applied. Two further models were tested, one that included fixed effects for market class assignments and another that included fixed effects for the first three principal component axes. A look at the QQ-plot showed no significant inflation of *p*-values when these fixed effects were excluded (results not shown), and no difference when they were introduced to the GWAS model. This can be attributed to the lack of population structure as reported previously, thus a model simply with a genomic relationship matrix was enough to avoid spurious associations between markers and traits. The threshold for identifying significant markers was −*log*_10_*p* = 3.65, after the 0.05 threshold was adapted for multiple testing ($\frac{0.05}{222}$). The signals detected when coding markers for additive or non-additive effects (in this case two levels of dominance) can be seen for dry matter and tuber count in Figure 5. Manhattan plots for tuber length and tuber weight are not shown as these plots were not very informative, however analyses on the significant markers for these traits still follow.

Across the five tested GWAS models for dry matter content, the most significant association with markers is observed when an additive effect is assumed (Figure 5). When compared to the plots assuming dominance we see that additivity gives both the highest scores [−*log*_10_(*p*)] and the most abundant markers appearing above the threshold. For tuber count we see significant markers in more abundance when a dominant coding of the marker matrix is considered. There are multiple flanking “hits” on chromosomes 1 and 3 when we assume simplex dominance for the reference allele. Chromosomes 4 (the two significant markers overlap on the plot) and 8 also show neighbouring markers with significant associations to tuber count when dominance is assumed to occur in the presence of a single alternative allele.

Manhattan plots of tuber weight did not show much evidence of significant QTLs in this analysis (Supplementary Figure 2). There are a few markers associated when dominant effects are modelled: these occur when the alternative allele is simplex or duplex dominant. Similarly, GWAS for tuber length analysis did not show any clear profile of associated markers (Supplementary Figure 2). Still we observed more significant markers when they were coded as duplex dominant for the alternative allele, however the highest scores were observed for additive effects and duplex dominance for the reference allele.

Manhattan plots can be ambiguous, therefore further analysis was done by performing a linear regression to uncover which marker effect type is more important for trait expression. Reported in Table 5 is the fit statistic for each regression (*R*^2^), which can be interpreted as the amount of phenotypic variance that can be explained by the significant markers and/or population structure.

**Table 5:** Percentage of variance explained (*R*^2^) from regression of each trait against: first three principal components only, significant additive markers after correcting for the first three principal components, significant dominance effect markers (under various configurations) after correcting for additive effects and the first three principal components.

**Trait**	**3 PCs only**	**3 PCs + Add markers**	**3 PCs + Add + Dom markers**			
			**1-dom-alt**	**1-dom-ref**	**2-dom-alt**	**2-dom-ref**
Tuber weight	5.55	12.92	12.26	12.26	30.40	16.88
Tuber count	0.07	12.50	35.92	14.99	23.43	15.78
Tuber length	6.13	42.43	53.24	44.68	57.75	47.73
Dry matter	41.77	67.01	67.96	66.64	68.49	66.24

Of the four traits, the variance of dry matter is best explained from marker information, and also has the biggest influence from principal components alone. This does not agree with previous population structure analysis (Figure 2), but those previous analyses were across the entire genome. For this trait, using only the significant additive markers found on chromosome 3, we can explain over 50% of phenotypic variation (not shown). For dry matter, the inclusion of dominance adds no information as seen in the GP results (Figure 4 and Table 5). Tuber count, as seen in GP, is controlled by dominance effects. This dominance effect comes from the alternative allele as opposed to the reference allele which was not clear from Figure 5, and more than doubles the explained phenotypic variance (12.50–35.92%) in comparison to additive effects. Explained phenotypic variation of tuber weight remains unchanged under simplex dominance assumptions. We see strong evidence that the level of dominance occurs at a duplex level, where the explained phenotypic variation increases from 12.92 to 30.40%, when compared to additive assumptions. A significant portion of the phenotypic variation of tuber length can be explained by additive effects (42%) and we do see an increase when dominance from the alternative allele is modelled (simplex or duplex), but this increase was not as noticeable as the increase shown when tuber count and weight are coded for dominant effects. In general the effect of population structure on explaining the variation of tuber traits (length, weight, and count) is small.

## Discussion

The primary focus of this study was to explore and compare statistical models for genomic prediction in tetraploid potato. As a secondary focus, a genome wide association analysis was conducted to identify trait architecture and thus explain the reasons for differences in prediction accuracies from trait to trait. The same marker profile was used for both GP and GWAS. It is worth noting that Bayes-R and Genome-wide Complex Trait Analysis (GCTA) can simultaneously perform GP and GWAS, therefore deserving of further study. However, for this study, we focused on a tetraploid species and therefore wanted to ensure that both the GP and GWAS analyses were tailored for tetraploids.

Translating these findings to a traditional breeding program expose the limitations of this study. Only 147 cultivars were analysed in this study, spread over several market classes. In a traditional breeding program thousands of new hybrids can be evaluated within one particular market class. Despite these limitations, the work done here still shows that there is merit using genomic selection, especially after the first round of phenotypic selection where a majority of the material has been discarded. After this stage genomic selection can then significantly speed up the breeding cycle.

### Heritability

Broad-sense Heritability estimates were quite high. As mentioned previously, our heritability estimates can more accurately be defined as repeatability estimates (Falconer et al., 1996), because we are averaging across six trials. Our high estimates show that there is not too much genotype by environment interaction (GxE) and thus high repeatability. The order of heritability estimates was unexpectedly not in agreement with the order of GP accuracies, and this was also found in a similar study (Stich and Van Inghelandt, 2018). The order of our heritability estimates is not a ranking of which traits would best be explained by marker information, although it does give some indication. Instead it is a ranking of which traits show the least to most GxE, with dry matter having the least GxE and tuber weight having the most. Regardless, one would expect heritability to translate to marker effects and GP accuracies not be so low in relation to heritability estimates.

### Genomic Prediction Models

For most traits, the differences between GP models were very small (Habyarimana et al., 2017; Sverrisdóttir et al., 2017; Amadeu et al., 2020). Amadeu et al. (2020) concluded that there is little difference in prediction accuracy between modelling strategies, and use of an additive GBLUP model would be sufficient for GP in auto-tetraploids. Only two traits in potato were analysed in that study: yield and specific gravity. Specific gravity is closely related to dry matter (Simmonds, 1977; Kumar et al., 2005), and in this study we also found that the additive GBLUP model is suitable. Tuber length also supports the conclusion by Amadeu et al. (2020), where a GBLUP additive model performs whole genome prediction as well as other models. For the other two traits analysed in this study, tuber count and yield, we have shown that other model considerations should be made to maximise prediction accuracy.

#### Capturing Dominance and Epistatic Effects

For tuber count, the modelling of dominance gave a 17% increase in prediction accuracy (from 0.35 to 0.41). The trait architecture revealed in the GWAS section, showed that significant dominant markers explained the most phenotypic variation, therefore targetting these non-additive effects resulted in the highest prediction accuracy. Trying to capture these non-additive effects with the full tetraploid model did not increase prediction accuracy.

Yield was one of the traits analysed by Amadeu et al. (2020), however, the RKHS model was not tested in that study. In this study, we show that the parametric models that included a term to capture epistasis did show evidence that there is an epistatic effect controlling tuber weight (which we consider as yield). The semi-parametric RKHS model produced the highest prediction accuracies for this trait. This is in agreement with findings from other studies that concluded epistasis is better captured by semi-parametric (and non-parametric) in comparison to parametric models (Howard et al., 2014; Jacquin et al., 2016; Momen et al., 2018). Based on GWAS results, there may be important dominance effects that were not explicitly modelled in our GP analyses of this trait. The GWAS results showed that, like tuber count, yield had the highest explained phenotypic variance when markers were coded as dominant. However, these markers that explained a significant portion of phenotypic variance for yield were coded as duplex dominant (instead of simplex). The dominance relation matrix used in our GP models assume simplex dominance, and there are no current adaptations to expand to higher levels of dominance (Amadeu et al., 2020).

The explicit modelling of epistasis for specific gravity in Endelman et al. (2018) gave a substantial increase in prediction accuracy, however that study also did not include the RKHS model. It would have been interesting to see if an RKHS model would have performed better, based on what we observed for epistasis in tuber weight and other previous studies as mentioned before. Interestingly, dry matter was not improved with the modelling of epistasis in this study, which contradicts the results of Endelman et al. (2018).

The full tetraploid model (Slater et al., 2016), developed to implicitly capture non-additive effects in auto-tetraploids, did not improve accuracies in this study and one other (Amadeu et al., 2020). In our analyses, this model performed least favourably for most traits. A possible reason for this is the use of genotype frequency instead of allele frequency. The marker data was dominated significantly with nulliplex and simplex dosages (>75% of information) and therefore genotype frequencies for other dosages may be severely under-represented. GBS data for tetraploid data has been said to bias against the alternate allele (Endelman, personal communication, November 07, 2019), which is most likely the cause of the imbalance of dosage classes for the data in this study. For this reason, it would be worthwhile to have another look at this model in a study with a more balanced marker profile.

#### Mixed Models (GBLUP) vs. Bayesian Models

Marker effect models are expected to perform better than mixed model GP models (GBLUP) when traits are controlled by a few high impact loci (de los Campos et al., 2013). Like the previous GP studies for potato (Habyarimana et al., 2017; Sverrisdóttir et al., 2017; Amadeu et al., 2020), this study also revealed very little difference between these two model classes. Despite the negligible differences, two traits did give some surprising results.

GWAS for dry matter found a few markers that were able to explain more than 50% of phenotypic variability. Therefore, for this trait, we would have expected a relevant increase going from a mixed model to marker effect model. Tuber yield showed a small but irrelevant increase (<0.03) when moving from mixed to marker effect modelling, however GWAS findings were unable to explain this result. The significant additive markers for yield explained very little phenotypic variation (12.92%), therefore it was unexpected that a marker effect model would have even slightly outperformed a GBLUP model. Habyarimana et al. (2017) also found that the Bayesian model gave more accurate predictions for yield than the traditional GBLUP model.

## Conclusions

- For GP in auto-tetraploids, there are very little differences between different types of shrinkage methods, and models that do both shrinkage and variable selection.
- GWAS can assist in deciding what model strategies should be considered, especially when considering capturing non-additive effects. When GWAS reveals significant dominant effect markers (simplex), this should be modelled specifically in GP models.
- Tuber weight shows evidence of epistasis, therefore semi- and non-parametric models should be used to predict this trait. Further investigation can include extending the dominance relationship matrix to include duplex dominance and modelling higher levels of epistasis.
- There is no one-size-fits-all model, especially when capturing non-additive effects. Understanding the nature of these effects, example dominance in tuber count vs. epistasis in tuber weight, is important information when choosing the most suitable model.

## Data Availability Statement

The original contributions presented in the study are included in the article/Supplementary Material, further inquiries can be directed to the corresponding author/s.

## Author Contributions

SW performed the analyses and drafted the manuscript. CZ developed the scripts for calculating genotype probabilities. CM, HM, and RV contributed to the discussion on analytical models and data preparation. AB performed bioinformatic analyses on the sequence data. FE guided analyses and was the general overseer for the project. All authors significantly contributed to the present study and read and approved the final manuscript.

## Conflict of Interest

The authors declare that the research was conducted in the absence of any commercial or financial relationships that could be construed as a potential conflict of interest.

## Publisher's Note

All claims expressed in this article are solely those of the authors and do not necessarily represent those of their affiliated organizations, or those of the publisher, the editors and the reviewers. Any product that may be evaluated in this article, or claim that may be made by its manufacturer, is not guaranteed or endorsed by the publisher.

## Supplementary Material

Supplementary Data Sheet 1 Phenotypic data.

Supplementary Data Sheet 2 Cross validation scheme.

Supplementary Data Sheet 3 Genotypic data.

Supplementary Data Sheet 4 Genotype probability details.

Supplementary Data Sheet 5 Supplementary figures.

## References

[B1] Amadeu R. R. Cellon C. Olmstead J. W. Garcia A. A. F. Resende M. F. R. Jr Muñoz P. R. (2016). AGHmatrix: R package to construct relationship matrices for autotetraploid and diploid species: A blueberry example. Plant Genome 9:plantgenome2016.01.0009. 10.3835/plantgenome2016.01.0009 27902800

[B2] Amadeu R. R. Ferrão L. F. V Oliveira I. B. Benevenuto J. Endelman J. B. Munoz P. R. (2020). Impact of dominance effects on autotetraploid genomic prediction. Crop Sci, 60. 10.2135/cropsci2019.02.0138

[B3] Annicchiarico P. Nazzicari N. Li X. Wei Y. Pecetti L. Brummer E. C. (2015). Accuracy of genomic selection for alfalfa biomass yield in different reference populations. BMC Genomics 16, 1020–1020. 10.1186/s12864-015-2212-y 26626170 PMC4667460

[B4] Ashraf B. H. Byrne S. Fé D. Czaban A. Asp T. Pedersen M. G.. (2016). Estimating genomic heritabilities at the level of family-pool samples of perennial ryegrass using genotyping-by-sequencing. Theor. Appl. Genet. 129, 45–52. 10.1007/s00122-015-2607-9 26407618 PMC4703624

[B5] Bernardo R. (1996). Best linear unbiased prediction of maize single-cross performance. Crop Sci. 36:cropsci1996.0011183X003600010009x. 10.2135/cropsci1996.0011183X003600010009x 24162487

[B6] Birch P. R. J. Bryan G. Fenton B. Gilroy E. M. Hein I. Jones J. T.. (2012). Crops that feed the world 8: potato: are the trends of increased global production sustainable? Food Secur. 4, 477–508. 10.1007/s12571-012-0220-1

[B7] Butler D. (2009). ASREML: ASREML() Fits the Linear Mixed Model. Hemel Hampstead: R Package Version 3.0.

[B8] Coombes N. E. (2009). Digger Design Search Tool in R. Available online at: http://nswdpibiom.org/austatgen/software/ (accessed July 10, 2020).

[B9] de Bem Oliveira I. Resende Marcio F. R. J. Ferro L. F. V. Amadeu R. R. Endelman J. B. Kirst M.. (2019). Genomic prediction of autotetraploids; influence of relationship matrices, allele dosage, and continuous genotyping calls in phenotype prediction. G3 9, 1189–1198. 10.1534/g3.119.400059 30782769 PMC6469427

[B10] de los Campos G. Hickey J. M. Pong-Wong R. Daetwyler H. D. Calus M. P. L. (2013). Whole-genome regression and prediction methods applied to plant and animal breeding. Genetics 193, 327–345. 10.1534/genetics.112.143313 22745228 PMC3567727

[B11] D'hoop B. B. Keizer P. L. C. Paulo M. J. Visser R. G. F. van Eeuwijk F. A. van Eck H. J. (2014). Identification of agronomically important qtl in tetraploid potato cultivars using a marker-trait association analysis. Theor. Appl. Genet. 127, 731–748. 10.1007/s00122-013-2254-y 24408376

[B12] D'hoop B. B. Paulo M. J. Mank R. A. van Eck H. J. van Eeuwijk F. A. (2008). Association mapping of quality traits in potato (*Solanum tuberosum* l.). Euphytica 161, 47–60. 10.1007/s10681-007-9565-5

[B13] Dufresne F. Stift M. Vergilino R. Mable B. K. (2014). Recent progress and challenges in population genetics of polyploid organisms: an overview of current state-of-the-art molecular and statistical tools. Mol. Ecol. 23, 40–69. 10.1111/mec.12581 24188632

[B14] Enciso-Rodriguez F. Douches D. Lopez-Cruz M. Coombs J. de los Campos G. (2018). Genomic selection for late blight and common scab resistance in tetraploid potato (*Solanum tuberosum*). G3 8, 2471–2481. 10.1534/g3.118.200273 29794167 PMC6027896

[B15] Endelman J. B. (2011). Ridge regression and other kernels for genomic selection with R package rrBLUP. Plant Genome 4, 250–255. 10.3835/plantgenome2011.08.0024

[B16] Endelman J. B. Carley C. A. S. Bethke P. C. Coombs J. J. Clough M. E. da Silva W. L.. (2018). Genetic variance partitioning and genome-wide prediction with allele dosage information in autotetraploid potato. Genetics 209, 77–87. 10.1534/genetics.118.300685 29514860 PMC5937173

[B17] Falconer D. S. D. S. Mackay T. F. C. Falconer D. Mackay T. F. (1996). Introduction to Quantitative Genetics, 4th Edn. Burnt Mill: Longman.

[B18] Gallais A. (2003). Quantitative Genetics and Breeding Methods in Autopolyploid Plants. Paris: Quae.

[B19] Garcia A. A. F. Mollinari M. Marconi T. G. Serang O. R. Silva R. R. Vieira M. L. C.. (2013). SNP genotyping allows an in-depth characterisation of the genome of sugarcane and other complex autopolyploids. Sci. Rep. 3:3399. 10.1038/srep03399 24292365 PMC3844970

[B20] Gianola D. van Kaam J. B. C. H. M. (2008). Reproducing kernel hilbert spaces regression methods for genomic assisted prediction of quantitative traits. Genetics 178, 2289–2303. 10.1534/genetics.107.084285 18430950 PMC2323816

[B21] Guo X. Cericola F. Fè D. Pedersen M. G. Lenk I. Jensen C. S. Jensen J. Janss L. L. (2018). Genomic prediction in tetraploid ryegrass using allele frequencies based on genotyping by sequencing. Front. Plant Sci. 9:1165. 10.3389/fpls.2018.01165 30158944 PMC6104567

[B22] Habyarimana E. Parisi B. Mandolino G. (2017). Genomic prediction for yields, processing and nutritional quality traits in cultivated potato (*Solanum tuberosum* l.). Plant Breed 136, 245–252. 10.1111/pbr.12461

[B23] Hickey J. M. Chiurugwi T. Mackay I. Powell W. Hickey J. M. Chiurugwi T.. (2017). Genomic prediction unifies animal and plant breeding programs to form platforms for biological discovery. Nat. Genet. 49:1297. 10.1038/ng.3920 28854179

[B24] Howard R. Carriquiry A. L. Beavis W. D. (2014). Parametric and nonparametric statistical methods for genomic selection of traits with additive and epistatic genetic architectures. G3 4, 1027–1046. 10.1534/g3.114.010298 24727289 PMC4065247

[B25] Inostroza L. Bhakta M. Acuña H. Vásquez C. Ibáñez J. Tapia G.. (2018). Understanding the complexity of cold tolerance in white clover using temperature gradient locations and a GWAS approach. Plant Genome 11. 10.3835/plantgenome2017.11.0096 PMC12810145 30512038

[B26] Jacquin L. Cao T.-V. Ahmadi N. (2016). A unified and comprehensible view of parametric and kernel methods for genomic prediction with application to rice. Front. Genet. 7:145. 10.3389/fgene.2016.00145 27555865 PMC4977290

[B27] Jansky S. (2009). Chapter 2 - breeding, genetics, and cultivar development, in Advances in Potato Chemistry and Technology, eds Singh J. Kaur L. (San Diego, CA: Academic Press), 27–62. 10.1016/B978-0-12-374349-7.00002-7

[B28] Jiang Y. Reif J. C. (2015). Modeling epistasis in genomic selection. Genetics 201, 759–768. 10.1534/genetics.115.177907 26219298 PMC4596682

[B29] Jombart T. Ahmed I. (2011). adegenet 1.3-1: new tools for the analysis of genome-wide SNP data. Bioinformatics 27, 3070–3071. 10.1093/bioinformatics/btr521 21926124 PMC3198581

[B30] Kumar D. Ezekiel R. Singh B. Ahmed I. (2005). Conversion table for specific gravity, dry matter and starch content from under water weight of potatoes grown in North-Indian plains. Potato J. 32, 79–84.

[B31] Li J. Ji L. (2005). Adjusting multiple testing in multilocus analyses using the eigenvalues of a correlation matrix. Heredity 95, 221–227. 10.1038/sj.hdy.6800717 16077740

[B32] Meuwissen T. H. Hayes B. J. Goddard M. E. (2001). Prediction of total genetic value using genome-wide dense marker maps. Genetics 157, 1819–1829. 10.1093/genetics/157.4.1819 11290733 PMC1461589

[B33] Momen M. Mehrgardi A. A. Sheikhi A. Kranis A. Tusell L. Morota G.. (2018). Predictive ability of genome-assisted statistical models under various forms of gene action. Sci. Rep. 8:12309. 10.1038/s41598-018-30089-2 30120288 PMC6098164

[B34] Nazareno A. G. Bemmels J. B. Dick C. W. Lohmann L. G. (2017). Minimum sample sizes for population genomics: an empirical study from an Amazonian plant species. Mol. Ecol. Resour. 17, 1136–1147. 10.1111/1755-0998.12654 28078808

[B35] Pembleton L. W. Cogan N. O. I. Forster J. W. (2013). Stampp: an r package for calculation of genetic differentiation and structure of mixed-ploidy level populations. Mol. Ecol. Resour. 13, 946–952. 10.1111/1755-0998.12129 23738873

[B36] Pérez P. de los Campos G. (2014). Genome-wide regression and prediction with the BGLR statistical package. Genetics 198, 483–495. 10.1534/genetics.114.164442 25009151 PMC4196607

[B37] Piepho H.-P. Williams E. Michel V. (2015). Beyond latin squares: a brief tour of row-column designs. Agron. J. 107, 2263–2270. 10.2134/agronj15.0144

[B38] Piepho H. P. (2009). Ridge regression and extensions for genomewide selection in maize. Crop Sci. 49, 1165–1176. 10.2135/cropsci2008.10.0595

[B39] R Core Team (2019). R: A Language and Environment for Statistical Computing. Vienna: R Foundation for Statstical Computing.

[B40] Rincent R. Laloë D. Nicolas S. Altmann T. Brunel D. Revilla P.. (2012). Maximizing the reliability of genomic selection by optimizing the calibration set of reference individuals: comparison of methods in two diverse groups of maize inbreds (*Zea mays* l.). Genetics 192, 715–728. 10.1534/genetics.112.141473 22865733 PMC3454892

[B41] Rosyara U. R. De Jong W. S. Douches D. S. Endelman J. B. (2016). Software for genome-wide association studies in autopolyploids and its application to potato. Plant Genome 9. 10.3835/plantgenome2015.08.0073 27898814

[B42] Simmonds N. W. (1977). Relations between specific gravity, dry matter content and starch content of potatoes. Potato Res. 20, 137–140. 10.1007/BF02360274

[B43] Slater A. T. Cogan N. O. I. Forster J. W. Hayes B. J. Daetwyler H. D. (2016). Improving genetic gain with genomic selection in autotetraploid potato. Plant Genome 9. 10.3835/plantgenome2016.02.0021 27902807

[B44] Stich B. Van Inghelandt D. (2018). Prospects and potential uses of genomic prediction of key performance traits in tetraploid potato. Front. Plant Sci. 9:159. 10.3389/fpls.2018.00159 29563919 PMC5845909

[B45] Su G. Christensen O. F. Ostersen T. Henryon M. Lund M. S. (2012). Estimating additive and non-additive genetic variances and predicting genetic merits using genome-wide dense single nucleotide polymorphism markers. PLoS ONE 7:e45293. 10.1371/journal.pone.0045293 23028912 PMC3441703

[B46] Sverrisdóttir E. Byrne S. Sundmark E. H. R. Johnsen H. Ø. Kirk H. G. Asp T.. (2017). Genomic prediction of starch content and chipping quality in tetraploid potato using genotyping-by-sequencing. Theor. Appl. Genet. 130, 2091–2108. 10.1007/s00122-017-2944-y 28707250 PMC5606954

[B47] VanRaden P. (2008). Efficient methods to compute genomic predictions. J. Dairy Sci. 91, 4414–4423. 10.3168/jds.2007-0980 18946147

[B48] Vos P. G. Paulo M. J. Voorrips R. E. Visser R. G. F. van Eck H. J. van Eeuwijk F. A. (2017). Evaluation of LD decay and various LD-decay estimators in simulated and snp-array data of tetraploid potato. Theor. Appl. Genet. 130, 123–135. 10.1007/s00122-016-2798-8 27699464 PMC5214954

[B49] Wallace J. G. Zhang X. Beyene Y. Semagn K. Olsen M. Prasanna B. M.. (2016). Genome-wide association for plant height and flowering time across 15 tropical maize populations under managed drought stress and well-watered conditions in Sub-Saharan Africa. Crop Sci. 56, 2365–2378. 10.2135/cropsci2015.10.0632

[B50] Whittaker J. C. Thompson R. Denham M. C. (2000). Marker-assisted selection using ridge regression. Genet. Res. 75, 249–252. 10.1017/S0016672399004462 10816982

[B51] Willing E.-M. Dreyer C. van Oosterhout C. (2012). Estimates of genetic differentiation measured by FST do not necessarily require large sample sizes when using many SNP markers. PLoS ONE 7:e42649. 10.1371/journal.pone.0042649 22905157 PMC3419229

[B53] Zheng C. Amadeu R. R. Munoz P. R. Endelman J. B. (2020). Haplotype reconstruction in connected tetraploid f1 populations. bioRxiv [Preprint]. 10.1101/2020.12.18.423519 PMC8633103 34849879

