# Supplementary material for: Understanding the Effectiveness of Genomic Prediction in Tetraploid Potato
Source: Front Plant Sci. 2021 Aug 9;12:672417. doi: 10.3389/fpls.2021.672417 (PMC8381724; doi:10.3389/fpls.2021.672417)
Supplement: Supplementary Data Sheet 4 — Genotype probability details. [file Data_Sheet_4.docx]

# Calculate genotype probabilities from GBS count data

Consider a single marker location within a given individual. Assume that the marker is bi-allelic, and denote by count data $y=(r_{1},r_{2})$ the numbers of reads for allele 1 and allele 2, respectively. One allele is mis-typed as the alternative allele with probability $\epsilon$.

Let z be the un-phased genotypes (also called dosage), that is, the number of allele 1 in the genotype. The aim is to infer the unknown z from the observed count data y. Specifically, we calculate the posterior probability $p(z|y, \epsilon)$ of dosage, conditional count data y and sequencing error probability $\epsilon$. According to the Bayesian rule,

$p\left( z | y,\epsilon\right)=\frac{p\left( y | z,\epsilon\right)p\left( z|\epsilon\right)}{p(y|\epsilon)}$ (1)

We assume that $\epsilon$ is known and drop the implicit dependence of probabilities on $\epsilon$. We have

$p\left( z | y \right)=\frac{p\left( y | z \right)p\left( z \right)}{p(y)}$ (2)

where the normalization constant $p\left( y \right)=\sum_{z=0}^{n} p\left( y | z \right)p\left( z \right)$. Here dosage z =0, ..., n for ploidy level n=2, ... In the following, we model the likelihood $p\left( y | z \right)$ and the prior $p\left( z \right)$.

**Likelihood**

Consider a non-transparent urn containing n alleles with the number z of allele 1 and the number n-z of allele 2. Draw one allele out of the urn (with replacement), it is allele 1 with probability z/n, and allele 2 with probability 1-z/n. Given the observation error $\epsilon$, the drawn allele is observed to be allele 1 with probability

$q_{1}=\frac{z}{n}\left( 1-\epsilon\right)+\left( 1-\frac{z}{n} \right)\epsilon$ (3)

where the first term on the right hand of side denotes the probability that the draw allele is allele 1 and it is observed without error. And the draw is observed to be allele 2 with probability $q_{2}=1-q_{1}=\left( 1-\frac{z}{n} \right)(1-\epsilon)+\frac{z}{n}\epsilon$

Assuming that reads are independent, the number of reads for allele 1 is binomially distributed with probability q1. Thus,

$$p\left[ y=\left( r_{1},r_{2} \right)|z \right]=\left( \begin{aligned} n \\ r_{1} \end{aligned} \right)\left( q_{1} \right)^{r_{1}}\left( 1-q_{1} \right)^{r_{2}}$$

We may ignore the binomial coefficient so that

$p\left[ y=\left( r_{1},r_{2} \right)|z \right]\propto\left( q_{1} \right)^{r_{1}}\left( 1-q_{1} \right)^{r_{2}}$ (4)

where $q_{1}=\frac{z}{n}+\left( 1-\frac{2z}{n} \right)\epsilon$.

Specifically for diploid n=2

$$p\left( r_{1},r_{2} | z=0,\epsilon\right)=\left( \begin{aligned} n \\ r_{1} \end{aligned} \right)\left( \epsilon\right)^{r_{1}}\left( 1-\epsilon\right)^{r_{2}}$$

 $p\left( r_{1},r_{2} | z=1,\epsilon\right)=\left( \begin{aligned} n \\ r_{1} \end{aligned} \right)\left( \frac{1}{2} \right)^{r_{1}}\left( \frac{1}{2} \right)^{r_{2}}$ (5)

$$p\left( r_{1},r_{2} | z=2,\epsilon\right)=\left( \begin{aligned} n \\ r_{1} \end{aligned} \right)\left( 1-\epsilon\right)^{r_{1}}\left( \epsilon\right)^{r_{2}}$$

where dosages z=0, 1, 2 corresponds to un-phased genotypes 22, 12, 11, respectively.

For tetraploid n=4

$$p\left( r_{1},r_{2} | z=0,\epsilon\right)=\left( \begin{aligned} n \\ r_{1} \end{aligned} \right)\left( \epsilon\right)^{r_{1}}\left( 1-\epsilon\right)^{r_{2}}$$

$$p\left( r_{1},r_{2} | z=1,\epsilon\right)=\left( \begin{aligned} n \\ r_{1} \end{aligned} \right)\left( \frac{1}{4}+\frac{1}{2}\epsilon\right)^{r_{1}}\left( \frac{3}{4}-\frac{1}{2}\epsilon\right)^{r_{2}}$$

 $p\left( r_{1},r_{2} | z=2,\epsilon\right)=\left( \begin{aligned} n \\ r_{1} \end{aligned} \right)\left( \frac{1}{2} \right)^{r_{1}}\left( \frac{1}{2} \right)^{r_{2}}$ (6)

$$p\left( r_{1},r_{2} | z=3,\epsilon\right)=\left( \begin{aligned} n \\ r_{1} \end{aligned} \right)\left( \frac{3}{4}-\frac{1}{2}\epsilon\right)^{r_{1}}\left( \frac{1}{4}+\frac{1}{2}\epsilon\right)^{r_{2}}$$

$$p\left( r_{1},r_{2} | z=4,\epsilon\right)=\left( \begin{aligned} n \\ r_{1} \end{aligned} \right)\left( 1-\epsilon\right)^{r_{1}}\left( \epsilon\right)^{r_{2}}$$

where dosages z=0, 1, 2, 3, 4 corresponds to un-phased genotypes 2222, 1222, 1122, 1112, 1111, respectively.

**Prior**

For simplicity, we have a discrete uniform distribution for the prior z,

$p\left( z \right)=1/(n+1)$ (7)

**Example**

Consider n=2, $\epsilon=0.001$, r1=0, and r2=1, q1=0.001,0.5, 0.999 for z=0, 1, 2

$$p\left( r_{1}=0,r_{2}=1 | z=0,\epsilon\right)=0.999$$

$$p\left( r_{1}=0,r_{2}=1 | z=1,\epsilon\right)=0.5$$

$$p\left( r_{1}=0,r_{2}=1 | z=2,\epsilon\right)=0.001$$

Since $p\left( z | y,\epsilon\right)\propto p\left( y=(0,1) | z,\epsilon\right)p\left( z|\epsilon\right)$

$$p\left( z=0 | y=\left( 0,1 \right),\epsilon\right)=\frac{0.999}{0.999+0.5+0.001}\approx0.67$$

$$p\left( z=1 | y=\left( 0,1 \right),\epsilon\right)=\frac{0.5}{0.999+0.5+0.001}\approx0.33$$

$$p\left( z=2 | y=\left( 0,1 \right),\epsilon\right)=\frac{0.001}{0.999+0.5+0.001}\approx0.00$$
