# Supplementary material for: Understanding the Effectiveness of Genomic Prediction in Tetraploid Potato
Source: Front Plant Sci. 2021 Aug 9;12:672417. doi: 10.3389/fpls.2021.672417 (PMC8381724; doi:10.3389/fpls.2021.672417)
Supplement: Supplementary Data Sheet 5 — Supplementary figures. [file Data_Sheet_5.docx]

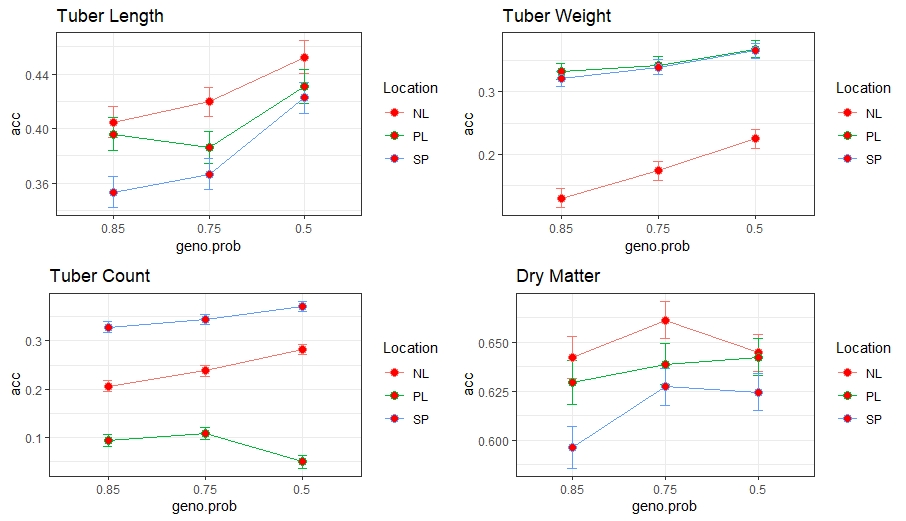


Supplementary Figure 1: Graphs of prediction accuracy versus genotype probability. The higher the genotype probability the less markers (0.85=19K markers, 0.75 =36K markers, 0.5 = 39K markers)


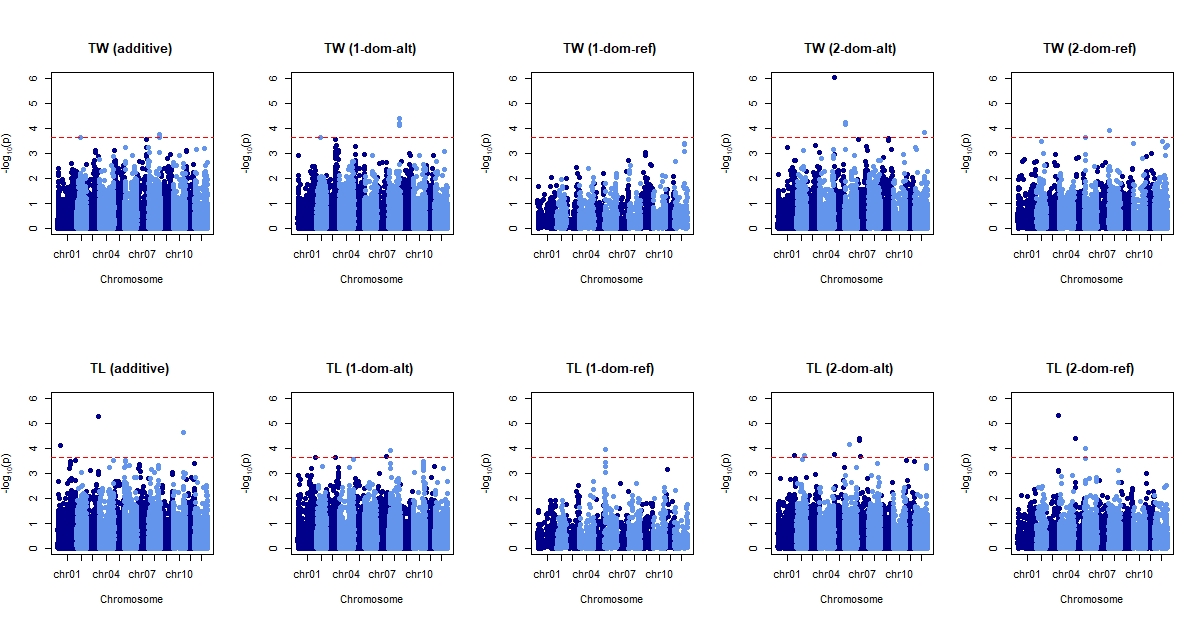


Supplementary Figure 2 : Manhattan Plots for Tuber Weight and Tuber Length
